# Endoscopic resection of a giant duodenal lipoma: a case report

**DOI:** 10.1055/a-2723-7557

**Published:** 2025-11-06

**Authors:** Ângela Domingues, Ricardo Araújo, Gonçalo Silva, Rita Sousa, Carlos Saraiva, Nuno Dias, Américo Silva

**Affiliations:** 1 679754 Gastroenterology Department, Unidade Local de Saúde Viseu Dão Lafões, Viseu, Portugal; 2 679754 Radiology Department, Unidade Local de Saúde Viseu Dão Lafões, Viseu, Portugal; 3 Patology Department, Unidade Local de Saúde Viseu Dão Lafões, Viseu, Portugal

59-year-old male patient underwent a screening endoscopy requested by his primary care physician, during which a duodenal lesion was identified.

The patient reported pain in the left upper quadrant.

Upper gastrointestinal endoscopy revealed a thick, finger-like polypoid lesion, originating from the proximal second portion of the duodenum (D2) and extending into the third portion (D3), with an approximate length of 10 cm.

EUS identified a duodenal lesion involving mucosa and submucosa, with retraction of the muscular layer in the proximal 2 cm.

Axial contrast-enhanced CT images showing an endophytic polypoid lesion in the second portion of the duodenum, with a central adipose and vascular component, extending toward the ligament of Treitz ( Video 1 ). Endoscopic ressection was performed, as demonstrated in the main video. Pathological examination reveald a duodenal lipoma ( Fig. 1 ).

Axial contrast-enhanced CT images showing an endophytic polypoid lesion in the second portion of the duodenum, with a central adipose and vascular component, extending toward the ligament of Treitz.Video 1

**Fig. 1:**
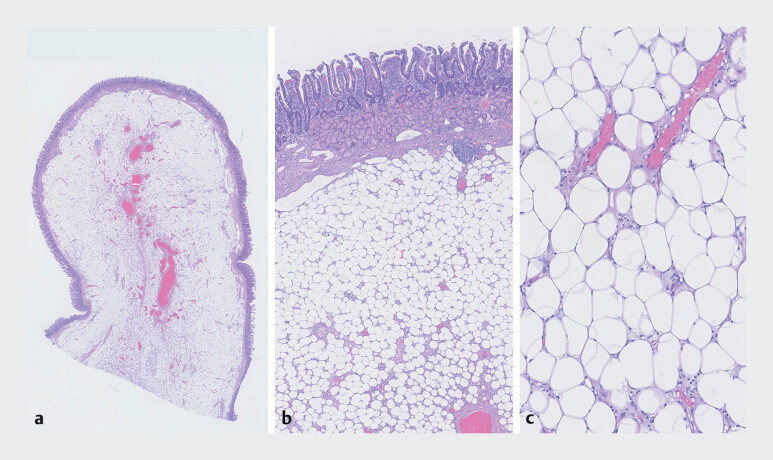
Duodenal mucosal and submucosal specimen without evidence of epithelial dysplasia, containing a submucosal lesion histologically consistent with a LIPOMA. **a** Polypoid submucosal lesion, low magnification. **b** Adipocytic proliferation in the duodenal submucosa (small intestinal mucosa with Brunner’s glands). **c** Mature unilocular adipocytes without easily identifiable lipoblasts or atypical stromal cells.

Lipomatous lesions of the gastrointestinal tract are rare and those of the duodenum are extremely unusual.

The majority is found incidentally, however, large duodenal lipomas may present with clinical symptoms such as abdominal pain, dyspepsia, intussusception and rarely, GI haemorrhage and iron deficiency anaemia.

The current recommendation is endoscopic excision, unless this is technically difficult and warrants surgical excision.

We present a rare case of giant lipomatous duodenal lesion, successfully treated with endoscopy.

Endoscopy_UCTN_Code_TTT_1AO_2AC

## Notes

Endoscopy E-Videos https://eref.thieme.de/e-videos
*E-Videos* is an open access online section of the journal *Endoscopy* , reporting on interesting cases and new techniques in gastroenterological endoscopy. All papers include a high-quality video and are published with a Creative Commons CC-BY license. Endoscopy E-Videos qualify for HINARI discounts and waivers and eligibility is automatically checked during the submission process. We grant 100% waivers to articles whose corresponding authors are based in Group A countries and 50% waivers to those who are based in Group B countries as classified by Research4Life (see: https://www.research4life.org/access/eligibility/ ). This section has its own submission website at https://mc.manuscriptcentral.com/e-videos .
